# Epidemiology of genital infections caused by *Mycoplasma hominis, M. genitalium* and *Ureaplasma urealyticum* in Iran; a systematic review and meta-analysis study (2000–2019)

**DOI:** 10.1186/s12889-020-08962-5

**Published:** 2020-06-29

**Authors:** Khadijeh Moridi, Mohammad Hemmaty, Amir Azimian, Mohammad Hosein Fallah, Hamid Khaneghahi Abyaneh, Kiarash Ghazvini

**Affiliations:** 1 Department of Microbiology and Virology, Faculty of Medicine, Mashhad University of Medical Sciences, Mashhad, Iran; 2 Antimicrobial Resistance Research Center, Buali Research Institute, Mashhad University of Medical Sciences, Mashhad, Iran; 3 Vice Chancellory for Health, Mashhad University of Medical Sciences, Mashhad, Iran; 4 Student Research Committee, Mashhad University of Medical Sciences, Mashhad, Iran; 5 Branch Razi Vaccine and Serum Research Institute, Agricultural Research, Education and Extension Organization (AREEO), Mashhad, Iran; 6 Salim Immune Production Co.Technology Incbator of Razi Vaccine and Serum Research Institute, Ahmad abad str, Mashhad, Iran; 7 Department of Microbiology, Faculty of Medicine, North Khorasan University of Medical Sciences, bojnurd, Iran; 8 Branch Razi Vaccine and Serum Research Institute, Agricultural Research, Education and Extension Organization (AREEO), Tehran, Iran; 9 Department of Food Hygiene and Quality control, Faculty of Veterinary Medicine, University of Tehran, Tehran, Iran

**Keywords:** *Mycoplasma hominis*, *M. Genitalium*, *Ureaplasma urealyticum*, Infertility, Iran

## Abstract

**Background:**

Although many species of mycoplasmas regard as normal flora, but some species causes serious genital disease. In Iran several epidemiological studies have documented the prevalence of *Mycoplasma hominis, M. genitalium* and *Ureaplasma urealyticum* in genital disorders. This meta-analysis is going to represent the prevalence of *M. hominis, M. genitalium* and *U. urealyticum* among Iranian couples and the correlation between mycoplasmas infection and infertility.

**Methods:**

We search online databases from January 2000 to June 2019. We used following MeSH keywords (Prevalence, *M. hominis, M. genitalium, U. urealyticum*, male, female, fertility, Infertility, genitourinary tract infection and Iran) with all possible combinations with “OR” and “AND”. Finally, forty-four articles from 2670 were chosen for data extraction and analysis by software using STATA version 14.0.

**Results:**

This meta-analysis revealed that the prevalence of *U. urealyticum* was 17.53% in Iran and the prevalence of *M. genitalium* and *M. hominis* were 11.33 and 9.68% respectively. The rate of *M. genitalium*, *M. hominis* and *U. urealyticum* infection in women with symptoms of genitourinary tract infection was higher than men with genitourinary tract infection (6.46% vs 5.4, 7.67% vs 5.88 and 21.04% vs 12.13%, respectively). As expected, the prevalence of *M. genitalium*, *U. urealyticum* and *M. hominis* among infertile women (12.73, 19.58 and 10.81%) were higher than fertile women (3%, 10. 85% and 4. 35%). Similarly, the prevalence of *M. hominis* and *U. urealyticum* among infertile men (14 and 21.18%) were higher than fertile men (4 and 3%). Based on this analysis, the rate of *U. urealyticum* was higher than *M. genitalium* and *M. hominis* among infertile men and women compared to the fertile group. The prevalence rate of *M. genitalium*, *M. hominis* and *U. urealyticum* in central provinces is higher than other parts of Iran.

**Conclusions:**

This meta-analysis reemphasizes a significant relationship between the infertility rate and *U. urealyticum*, *M. genitalium* and *M. hominis* infections. Our finding help to plan the prevalence map of *M. hominis, M. genitalium* and *U. urealyticum* in Iran but further studies are needed to suggest routine screening of the pathogens.

## Background

Mycoplasma and Ureaplasma geniuses are the smallest self-replicating organism that belong to the Mollicutes class [1–4]. They live as external parasites of the human, animal, bird, insect and plant cells. Some species have a free-living existence in soil and water [5]. Since Dienes and Edsall isolated first mycoplasma from human in a Bartholin’s gland abscess in 1937, seventeen species of human mycoplasmas species have been identified [6, 7]. As a new derivative genus Ureaplasma is divided in to 14 known serotypes and two biovars: *U. parvum* and *U. urealyticum*. *U. urealyticum* can be transmitted in different ways, including directly by sexual transmission, vertically from mother to offspring, or through transplanted tissues [8–13]. Generally, genital mycoplasmas such as *M. hominis, M. genitalium* and *U. urealyticum* are important emerging sexually transmitted bacterial pathogens capable to cause asymptomatic, long-term and chronic infection in genitourinary tract which is considered to be a threat to community health [14, 15]. In a clinical study, about 40% of infants born from infected mothers with genital Mycoplasma infection had symptomatic infection such as neonatal conjunctivitis and meningitis by an ascending route or by crossing the placenta from the mother’s blood via delivery through a colonized birth canal [16].

Despite the worldwide incidence of genital mycoplasmas infections, there are no accurate reports of prevalence, common types, common routes of transmission and antibiotic resistance patterns of *M. genitalium, M. hominis* and *U. urealyticum* in Iran [17]. There are some studies about the presence of genital mycoplasmas among men, women, pregnant, newborns, infertile and *etc* in Iran. In this systematic review and meta-analysis, we are going to present an illustration of prevalence of *M. hominis, M. genitalium,* and *U. urealyticum* in Iran and the correlation between mycoplasmas infection and infertility in Iranian couples.

## Methods

### Search strategy

We search online databases including Pubmed, Scopus, Science Direct, IranMedex, SID (Scientific Information Database), and Google Scholar for the papers that were performed in Iran from January 2000 to June 2019. We used following MeSH keywords (Prevalence, *M. hominis, M. genitalium, U. urealyticum*, male, female, fertility, Infertility, genitourinary tract infection and Iran) with all possible combinations with “OR” and “AND”. Then the titles of the articles were entered into Mendeley software to find similar articles. Difinition of terms were considered as WHO recommended. One of the limitations of this study is the lack of data in some part of Iran. Since different researchers worked on different samples and conditions, the data was categorized in six groups: 1. Fertile men 2. Infertile men 3. Men with urinary tract infection or prostatitis 4. Fertile women 5. Infertile women 6. Women with urogenital infection or abortion or pregnant.

### Inclusion and exclusion criteria

Inclusion criteria of this study consisted of a reference to the prevalence of *M. genitalium, M. hominis,* and *U. urealyticum* in Iranian men and women by culture and PCR. Exclusion criteria were irrelevance or limited information, countries other than Iran, review articles, methods other than culture and PCR. At the end, 44 articles, which met our inclusion criteria, were conducted for meta- analysis.

### Data extraction

The data were extraction by a pre-prepared checklist from all included articles. The checklist included the author’s name, year of the study, the location, sample volume, type of specimen and the prevalence of *M. genitalium, M. hominis*, and *U. urealyticum.* The studies on each Mycoplasma species were further categorized into subgroups, considering (1) study population according to gender (men and women) fertile, infertile and urogenital tract infection; (2) Analytical method (including PCR, and culture); (3) geographical region of sampling (including **Eastern provinces**: Kerman, North Khorasan, Razavi Khorasan, South Khorasan, Sistan and Baluchestan, and Yazd Provinces; **Middle provinces** (Northern, Central & Southern): Alborz, Golestan, Mazandaran, Qazvin, Qom, Semnan, Tehran, Bushehr, Chaharmahal and Bakhtiari, Fars, Hormozgan, Isfahan, Kohgiluyeh and Boyer-Ahmad Provinces; **Western provinces**: Ardabil, East Azerbaijan, Gilan, Kordestan, West Azerbaijan, Zanjan, Hamadan, Ilam, Kermanshah, Khuzestan, Lorestan and Markazi Provinces).

### Analytic approach

The ratio of positive samples to total samples was defined as prevalence. Meta-analysis was conducted by STATA version 14 for prevalence of each bacterium on available data. Chi-squared (Q) and I-squared tests were used to assess heterogeneity among the studies. Since the heterogeneity was statistically significant (*p*-value of Q test < 0.1 and *I*^*2*^ index > 75%), a random-effects model was used; The outcome was estimated as prevalence and 95% confidence intervals (CI).

## Results

### Description of included and excluded studies

**Fig. 1:**
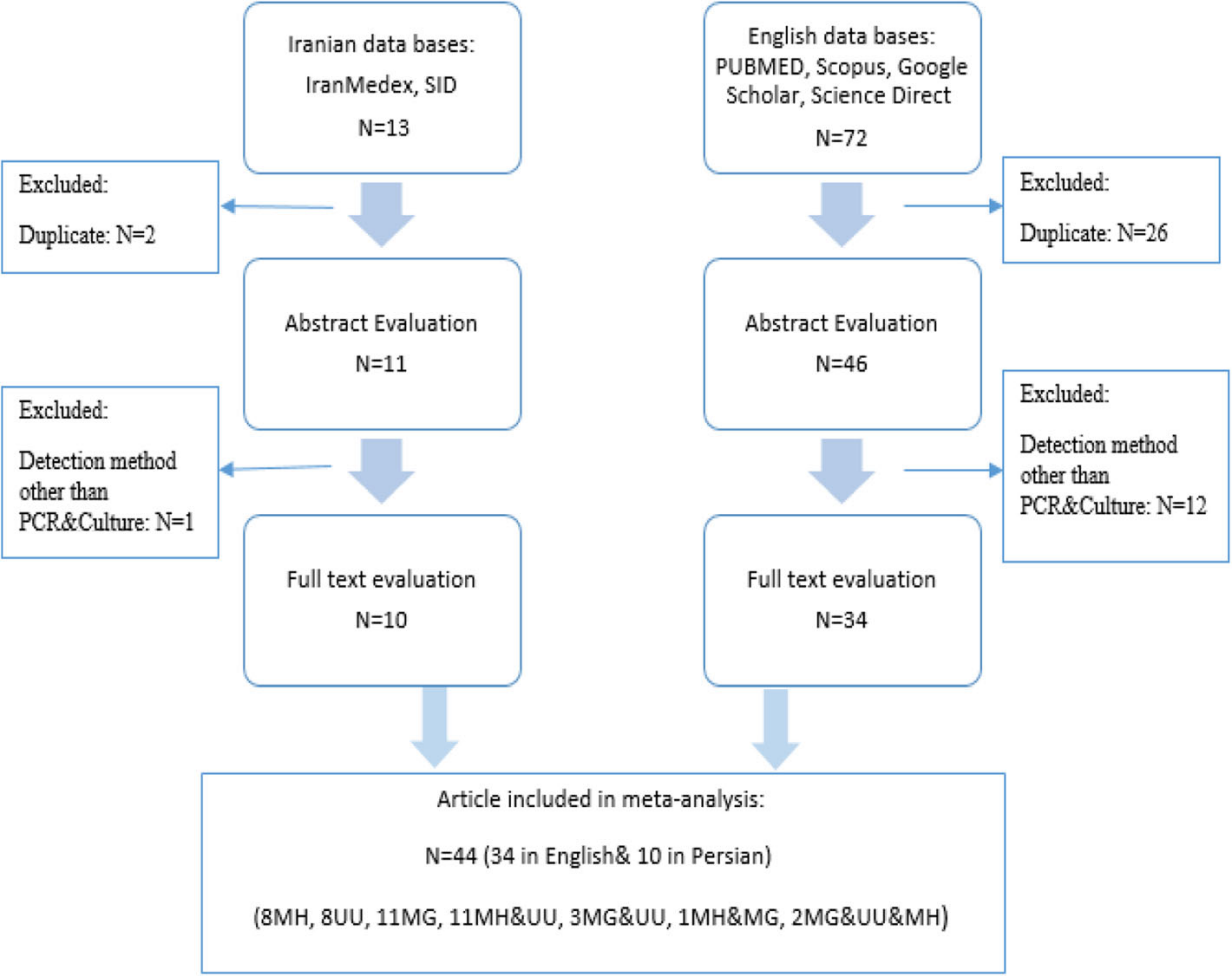
Flow chart of the literature search, systematic review and study selection

Initially 11,345 articles were identified through database searching. About 2670 articles were remained after discarding duplicate papers based on title and abstract. From 2670 articles, we excluded further 1606 papers based on exclusion criteria (489 papers on *M. genitalium*, 595 papers on *M. hominis*, and 522 papers on *U. urealyticum* were excluded). Forty-four original articles (full texts) related to prevalence of *M. genitalium*, *M. hominis*, and *U. urealyticum* in Iranian men and women in our literature review remained for reviewing and assessing for eligibility criteria. The final 44 articles were included: *M. genitalium* [17], *M. hominis* [18], *U. urealyticum* [19] with some of them contains two [15] or three [2] of these bacteria (Fig. 1). Table 1 provides an overview of the eligible studies.

**Table 1:** Prior studies concerning prevalence of *Mycoplasma hominis*, *Mycoplasma genitalium* and *Ureaplasma urealyticum* in Iran

no:	Location	Year	Author	Number & kind of sample	Method	Prevalence (%)	Comment	Ref
1	Tehran	2001	Badami	*n* = 375 cervical swab	Culture	Fertile women: MH = 18(7.2) & UU = 48(19.2) Infertile women: MH = 32(25.6) & UU = 41(32.8)	Fertile women = 250 Infertile women = 125	[20]
2	Tehran	2003	Salari	*n* = 125 swab urethral	PCR	MG = 9(7.2) UU = 24(19.2) MH = 3(2.4)	Men with NGU	[21]
3	Tehran	2005	AleYasin	*n* = 312 cervical swab	Culture PCR	Culture: MH = 20(6.4) PCR:MH = 50(16) Culture &PCR = 16(5)	Infertile women	[22]
4	Tehran	2005	Najar Peerayeh	*n* = 312 cervical swab	Culture PCR	Culture: UU = 18(5.7) PCR: UU = 32(10.25)	Infertile women	[23]
5	Tehran	2006	Najar Peerayeh	*n* = 377 cervical swab	PCR	MH = 31(8.2) UU = 60(15.9) MH & UU = 25(6.6)	Infertile women	[18]
6	Tehran	2007	Golshani	*n* = 200 semen samples	Multiplex PCR	MH = 22(11) UU = 6(3) MH&UU = 2(1)	Infertile men	[24]
7	Tehran	2007	Zeighami	*n* = 200 semen samples	PCR	Fertile men: UU = 3(3) Infertile men: UU = 12(12)	Fertile men = 100 Infertile me*n* = 100	[19]
8	Tehran	2007	soleimani rahbar	*n* = 100 semen samples	PCR	MH = 3(3) UU = 17(17)	Infertile men	[25]
9	Tehran	2007	Najar Peerayeh	*n* = 377 cervical swab	PCR	UU = 85(22.5)	Infertile women	[26]
10	Tehran	2008	NajarPeerayeh	*n* = 312 cervical swab	Culture PCR	Culture: MH = 12(4) & UU = 39(12) PCR: MH = 28(9) & UU = 54(17)	Infertile women	[27]
11	Tehran	2008	Ghazisaidi	*n* = 75 urethral secretion samples after prostatic massage & First void urine	PCR	urethral secretion: MH = 11(15) & UU = 19(25) First void urine: MH = 9 (12)& UU = 17(23)	Men suffering from nongonococcal urethritis and non-specific urethritis	[28]
12	Tehran	2008	Najar Peerayeh	*n* = 246 semen samples	PCR	Fertile men: UU = 3(3) Infertile men: UU = 23(15.7)	Fertile men = 100 Infertile men = 146	[29]
13	Tehran	2009	Amirmozafari	*n* = 210 cervical swab	Culture PCR	Culture: UU = 69(32.8) PCR: UU = 67(31.9)	Women with urogenital infection	[30]
14	Tehran	2010	Ahmadi	*n* = 220 semen sample	PCR	MH = 34 (15) UU = 89(40) MH&UU = 25(11)	Infertile men	[31]
15	Tehran	2011	Mirnejad	*n* = 210 genital samples	PCR	UU = 89(42.4) MG = 7(3.3)	Women with urogenital infection	[32]
16	Sabzevar	2011	Haghighi Hasanabad	*n* = 196 urine	PCR	MG = 2(1)	Pregnant women	[33]
17	Ahwaz	2011	Moosavian	*n* = 265 urine = 110 cervical swab = 155	Culture Multiplex PCR	Culture: MH = 5 (1.8)& UU = 0 Multiplex PCR: MH = 11(4)&UU = 13(5)	Women with urogenital infection	[34]
18	Kerman	2013	Vosooghi	*n* = 58 semen sample	PCR	MH = 13(22)	Infertile men	[35]
19	Tehran	2013	irajian	*n* = 200 paraffin blocks	PCR	MG = 4(2)	Men with prostatitis	[36]
20	Ahvaz	2013	Maleki	*n* = 265 urine = 110 cervical swab = 155	Multiplex PCR	Urine: MH = 11(10) & UU = 13(12) cervical swab: MH = 7 (4)& UU = 15(10)	Women with urogenital infection	[37]
21	Tehran	2013	Yeganeh	*n* = 200 urine	PCR	MG = 14(7)	Men refer to urology clinic	[38]
22	Tehran	2013	Sadrpour	*n* = 120 semen samples	PCR	MG = 12(10)	Infertile men	[39]
23	Mazandaran	2013	mohseni	*n* = 44 genital samples	PCR	MG = 10(22.7)	Pregnant women	[40]
24	Tehran	2014	Seifoleslami	*n* = 350 cervical swab	PCR	Infertile women: MH = 8(5.3) & UU = 10(6.6) MH&UU = 4(2.6) Fertile women: MH = 3(1.5) & UU = 5(2.5) MH&UU = 1	Infertile women = 150 Fertile women = 200	[17]
25	Kurdistan	2014	Ahmadi	*n* = 218 cervical swab	PCR	Pregnant women: UU = 8(7.3) Spontaneous abortion: UU = 18(16.5)	Pregnant women = 109 Spontaneous abortion = 109	[41]
26	Sanandaj	2014	Mousavi	*n* = 104 cervical swab	Multiplex PCR	MH = 3(3) MG = 3(3) UU = 39(37) MH&UU = 1(1) MH&MG = 1(1) MG&UU = 1(1) MG&UU&MH = 1(1)	Infertile women	[42]
27	Kerman	2014	JamalizadehBahaabadi	*n* = 200 semen sample = 100 cervical swab = 100	PCR	Semen sample: MH = 15(7) cervical swab: MH = 18(18)	Infertile men & Infertile women	[43]
28	Kerman	2014	Mohseni Moghadam	*n* = 200 semen samples = 100 cervical swab = 100	PCR	Semen samples: MG = 13(13) cervical swab: MG = 10(10)	Infertile men Infertile women	[11]
29	Tehran	2014	Sobouti	*n* = 330 cervical swab their baby after delivery	Multiplex PCR	Pregnant women: MH = 25(15.1) & UU = 25(15.1) their baby after delivery: MH = 15(9) & UU = 18(10.9)	Pregnant women = 165 their baby after delivery = 165	[44]
30	Tehran	2015	Dadashi	*n* = 124 sample from ovarian cancer	PCR	MG = 9(7.2)	Ovarian Cancer = 62 Benign Ovarian Cancer =62	[45]
31	Tehran	2015	Eslami	*n* = 124 paraffin blocks	PCR	MG = 0 UU = 1(0.8)	From men who undergo prostatectomy	[46]
32	Tehran	2015	Safavifar	*n* = 45 semen samples	PCR	Infertile men: MG = 6(40) Fertile men: MG = 11(37)	Infertile men = 15 Fertile men = 30	[47]
33	Kerman	2015	EftekhariMoghadam	*n* = 50 urine	PCR	MH = 3(6)	UTI patients	[14]
34	Kashan	2015	Safari	*n* = 864 urine	PCR	MH = 1(0.1)	UTI patients	[48]
35	Sanandaj	2016	Ramazanzadeh	*n* = 218 genital swab	PCR	Normal pregnant: MG = 4(3.6) Spontaneous abortion: MG = 2(1.8)	Normal pregnant = 109 Spontaneous abortion = 109	[49]
36	Tehran	2016	Ahmadi	n = 330 semen samples	Real-time PCR	Infertile men = MH = 24(14) Fertile men: MH = 6(4)	Infertile men = 165 Fertile men = 165	[50]
37	Qazvin	2016	Bahrami	*n* = 232 cervical swab	culture	UU = 87(37.5)	married females(20–50 years)	[51]
38	Qom	2016	Asgari	*n* = 187 semen samples	PCR	MH = 71(39)	Infertile men	[52]
39	Tehran	2016	Irajian	*n* = 200 prostatitis tissues with paraffin	PCR	UU = 7(3.5)	men suffering from prostatitis	[53]
40	Tehran	2017	sameni	*n* = 65 cervical swab	PCR	MG = 11(16.9)	Infertile women	[54]
41	Tehran	2017	Javadinia	*n* = 194 urine	PCR	UU = 22(11.3) MG = 11(5.6) UU&MG = 5(2.6)	Pregnant women	[55]
42	Mashhad	2017	Makari golkhatmi	*n* = 200 vaginal swab	PCR-ELISA	Infertile women: MG = 21(21) Fertile women: MG = 3(3)	Infertile women = 100 Fertile women = 100	[56]
43	Hamedan	2018	Moradi	*n* = 234 cervical swab	Culture PCR	Culture: MH = 14(5.9) PCR: MH = 30(12.8)	married females(20–50 years)	[57]
44	Mashhad	2018	Moridi	*n* = 100 semen samples	Culture PCR	Culture: MH = 7(7) PCR: MH = 8(8) Culture: MG = 0(0) PCR: MG = 0(0)	Infertile men	^a^

^a^The data is under publication

### Prevalence of *M. genitalium*

The overall prevalence of *M. genitalium* was 16.60% (CI 95%; 12.01–21.18%) and 8.26%(CI 95%; 6.33–10.19%) in male and female respectively (Table 2).

**Table 2:** The prevalence of *M. genitalium* in Iran based of meta-analysis

Study Population	studies	sample	prevalence, 95% CI	Model
**Men**	8	1114	16.60, 12.01–21.18	Random
*Fertile*	1	30	37.00, 36.83–37.17	Random
*Infertile*	4	435	21.00, 13.18–28.82	Random
*Symptomatic*^*1*^	4	649	5.40, 1.55–9.25	Random
**Women**	11	1455	8.26, 6.33–10.19	Random
*Fertile*	1	100	3.00, 2.97–3.03	Random
*Infertile*	4	369	12.73, 4.44–21.01	Random
*Symptomatic*^*2*^	7	986	6.46, 4.62–8.29	Random
**Women and men**	*18*	2569	11.33, 9.58–13.08	Random

*1. Men with urinary tract infection or prostatitis; 2. Women with urogenital infection or abortion or pregnant*

### Prevalence of *M. hominis*

The overall prevalence of *M. hominis* was 10.73% (CI 95%; 6.77–14.69%) and 8.83% (CI 95%; 6.67–10.98%), among male and female respectively (Table 3).

**Table 3:** The prevalence of *M. hominis* in Iran based of meta-analysis

Study Population	studies	sample	prevalence, 95% CI	Model
**Men**	12	2344	10.73, 6.77–14.69	Random
*Fertile*	1	165	4.00, 3.97–4.03	Random
*Infertile*	7	1130	14.00, 7.45–20.55	Random
*Symptomatic*^*1*^	4	1049	5.88, 2.18–9.57	Random
**Women**	12	3670	8.83, 6.67–10.98	Random
*Fertile*	2	450	4.35, −1.24 - 9.94	Random
*Infertile*	7	1480	10.81, 7.18–14.45	Random
*Symptomatic*^*2*^	3	1740	7.67, 4.34–10.99	Random
**Women and men**	*22*	6014	9.68, 7.75–11.61	Random

*1. Men with urinary tract infection or prostatitis; 2. Women with urogenital infection or abortion or pregnant*

### Prevalence of *U. urealyticum*

The prevalence of *U. urealyticum* was 13.92% (CI 95%; 7.58–20.26%) and 19.43% (CI 95%; 11.56–27.30%), in male and female respectively (Table 4).

**Table 4:** The prevalence of *U. urealyticum* in Iran based of meta-analysis

Study Population	studies	sample	prevalence, 95% CI	Model
**Men**	8	1290	13.92, 7.58–20.26	Random
*Fertile*	2	200	3.00, 2.98–3.02	Random
*Infertile*	4	766	21.18, 8.61–33.74	Random
*Symptomatic*^*1*^	4	324	12.13, 3.23–21.02	Random
**Women**	14	4441	19.43, 11.56–27.30	Random
*Fertile*	2	450	10.85, −5.52 - 27.22	Random
*Infertile*	7	1757	19.58, 13.59–25.57	Random
*Symptomatic*^*2*^	5	2234	21.04, 8.95–33.13	Random
**Women and men**	*24*	5731	17.53, 11.40–23.66	Random

*1. Men with urinary tract infection or prostatitis; 2. Women with urogenital infection or abortion or pregnant*

The prevalence rates of genital mycoplasma infection are due to *U. urealyticum*, *M. genitalium* and *M. hominis* respectively, in Iran. This study shows that the rate of *U. urealyticum*, *M. genitalium* and *M. hominis* infection in women with symptoms of genitourinary tract infection was higher than men with genitourinary tract infection. The result indicated that the prevalence of *U. urealyticum, M. genitalium* and *M. hominis* in infertile women were higher than fertile women. However, the prevalence of *U. urealyticum* and *M. hominis* in infertile men were higher than fertile men.

### Geographical distribution of *M. hominis, M. genitalium* and *U. urealyticum* in Iran

In Eastern provinces of Iran, the prevalence of *M. genitalium* and *M. hominis* were 9.60 and 9.73% respectively based of meta-analysis (CI 95%). There is no documented study on *U. urealyticum* in Eastern provinces. In Middle provinces, the prevalence of *M. genitalium*, *M. hominis* and *U. urealyticum* were 13.39, 11.17 and 17.94% respectively. While in Western provinces of Iran, the prevalence of *M. genitalium*, *M. hominis* and *U. urealyticum* were 3.3, 5.65 and 14.98% respectively (Table 5).

**Table 5:** The prevalence and 95% CI of *Mycoplasma hominis, Mycoplasma genitalium and Ureaplasma urealyticum* in different regions of Iran based of meta-analysis

Study Location	*M. genitalium*		*M. hominis*		*U. urealyticum*	
	provinces (studies)	prevalence, 95% CI	provinces (studies)	prevalence, 95% CI	provinces (studies)	prevalence, 95% CI
**Eastern provinces**^**1**^	2 (4)	9.60, 4.01–15.19	2 (5)	9.73, 4.49–14.96	0	–
**Middle provinces**^**2**^	2 (12)	13.39, 11.14–15.64	2 (11)	11.17, 8.10–14.24	2 (17)	17.94, 11.08–24.80
**Western provinces**^**3**^	1 (2)	3.30, 2.71–3.89	3 (4)	5.65, 3.09–8.22	2 (4)	14.98, 6.83–23.12

(1) ***Eastern provinces****: Kerman, North Khorasan, Razavi Khorasan, Sistan and Baluchestan, South Khorasan and Yazd Provinces;* (2) ***Middle provinces****: (Northern Central & southern): Alborz, Golestan, Mazandaran, Qazvin, Qom, Semnan, Tehran, Bushehr, Chaharmahal and Bakhtiari, Fars, Hormozgan, Isfahan, Kohgiluyeh and Boyer-Ahmad Provinces;* (3) ***Western provinces****: Ardabil, East Azerbaijan, Gilan, Kordestan, West Azerbaijan, Zanjan, Hamadan, Ilam, Kermanshah, Khuzestan, Lorestan and Markazi Provinces*

### Analytical method

**Fig. 2:**
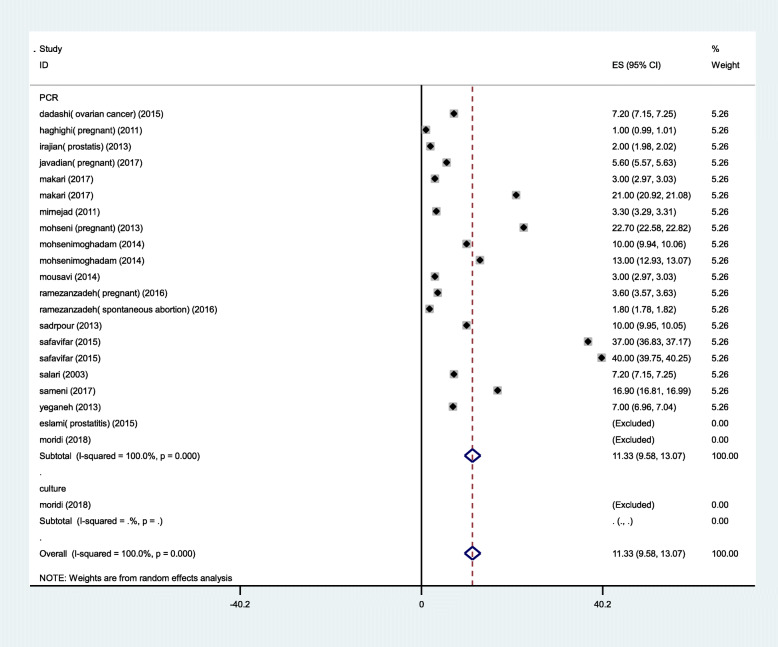
Forest plot of the meta-analysis of the prevalence of *M.genitalium* in Iran based on analytical method

**Fig. 3:**
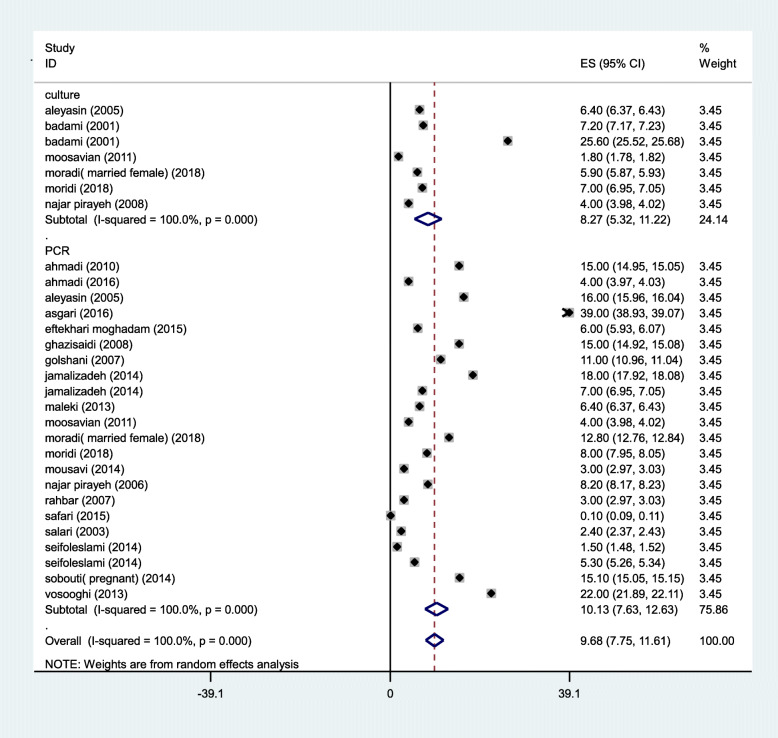
Forest plot of the meta-analysis of the prevalence of *M. hominis* in Iran based on analytical method

**Fig. 4:**
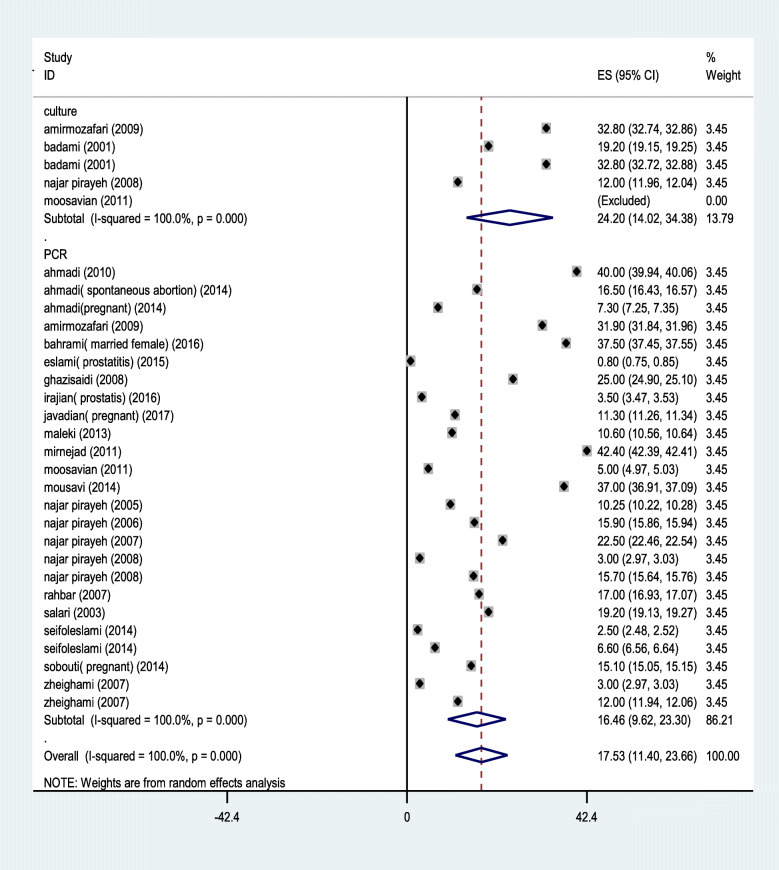
Forest plot of the meta-analysis of the prevalence of *U. urealyticum* in Iran based on analytical method

The forty- four selected articles which met our inclusion criteria were analyzed according to the culture and PCR methods. The prevalence rate of *M. hominis* and *M. genitalium* base on PCR (10.13%&11.33%) was higher than culture method (8.27%& 0%), whereas that was contrary in *U. urealyticum* (Figs. 2, 3 and 4).

## Discussion

The epidemiology and role of *M. hominis, M. genitalium* and *U. urealyticum* in infertility has been less discussed in Iran [14]. The different reports documented in other countries around the world. *M. genitalium* has been identified as a causative agent of 10–35% nongonococcal-nonchlamydia urethritis [58–62]. According to the community-based prospective cohort study from Oakeshott (2010) *M. genitalium* is found in 0.7 to 3.3% of women in general populations, while the prevalence in high-risk groups such as sex workers and STD clinic attendees is 7–22% in London [5, 63]. However, *M. hominis* resides commensally on the mucosal surfaces of the cervix or vagina. It’s colonization values ranges between 20 and 30% around the world [48, 64]. *M. hominis* was detected in 21–53% of women without genitourinary tract infection and at a lower percentage in the urethra of male [1]. Several studies have proposed that *M. hominis* is potentially pathogenic and sometimes associated with a variety of disorders including bacterial vaginosis, pyelonephritis, pelvic inflammatory disease, chorioamnionitis, endometritis, preterm birth, low birth, spontaneous abortion, stillbirth, premature birth, postpartum fever, perinatal mortality and infertility overtime [65, 66]. The positive rates of *M. hominis*, *M. genitalium* and *U. urealyticum* are controversial and diverse in the world [67]. Recently, Ghadiri (2019) in Iran (Ahwaz) detected *U. urealyticum* (28%) and *M. hominis* (10%) in semen specimens of infertile men by PCR and isolated 22% of *U. urealyticum* and 2% of *M. hominis* in the same samples by culture. While, *U. urealyticum* and *M. hominis* were detected in 50% & 26% by PCR of endocervical swabs specimens of infertile women and 8% & 4% by culture [68]. Christian Leli (2018) was detected *U. urealyticum* in 4.7%, *M. hominis* in 3.4% and *M.genitalium* in 0% of 232 cervical swab specimens of infertile women by real-time PCR in Italy [69]. Xiaofei Zhu (2016) showed that the prevalence of *U. urealyticum* and *M. hominis* were 42.3 and 0.4% among 7374 infertile men by culture [70]. Mahlangu (2019) was determined *M. genitalium* in 8.9% of urine and 10.6% of endocervical swab specimens which collected from males and females with genital discharge syndrome [71].

Baumann (2017) performed a meta-analysis on prevalence of *M. genitalium* and found that: the prevalence among women is similar to men and was 1.4% in developed countries and 3.9% in developing countries among general population. He showed that the prevalence among pregnant women were 0.9%, and the prevalence among men who have sex with men in the community was 3.2%, and among female commercial sex workers was 15.9% in the world [72]. Huang performed meta-analysis study (2015) and investigated the association between *U. urealyticum*, and *M. hominis* positive rate (5.2% & 14.9%) and risk of male infertility. While the *M. genitalium* prevalence did not showed any correlation to male infertility [73]. Kasprzykowska (2018) indicated that the prevalence of Ureaplasma *spp* in women (14.4%) and men (3.9%) is higher than *M. hominis* in women (0.2%) and men (0.2%) with urogenital tract infection in Poland [74]. Cassell estimated that the *U. urealyticum* can be found in 40 to 80% of cervicovaginal samples from sexually mature women [74, 75]. Zinzendorf (2008) investigated *M. hominis* in 23.8% of infertile men in Africa [76]. Taken (2016) could determine *M. hominis* in 3% of infertile men in Turkey [77]. Abusarah (2013) detected *U. urealyticum* in 10.8% versus 5.7% and *M. genitalium* in 3.2% versus 1.4% among infertile and fertile men respectively in Jordan [78]. Jensen indicated *M. genitalium* in 17% of male patient with urogenital tract infection in Denmark [79]. Al- Sweih (2012) in Kuwait detected *M. hominis* in 17.1% & 32.4%, *M. genitalium* in 4.7% & 3.2% and *U. urealyticum* in 24.4% & 26.1%, among infertile and fertile men respectively [80]. Lee (2013) displayed *U.urealyticum* in 48% & 25%, *M. hominis* in 14% & 6.3% of infertile and fertile men, while, *U. urealyticum* in 40% & 22.9% and *M. hominis* in 8% & 4.2% of infertile and fertile women in Korea [81]. Andersen reported that the prevalence of infection due to *M. genitalium* in general population was 2.3% in women and 1.1% in men whereas that was about 19% in men with urethritis and 11% in women with cervicitis in Denmark [82]. Also Grześko indicated *M. genitalium* from 19.6% of specimens obtained from cervical canal of infertile women, whereas it was 4.4% in control group (women with proven fertility) in Poland [83].

The prevalence rates of Mycoplasma and Ureaplasma are not well established and varies from one study to another. The heterogeneity of prevalence of mycoplasma urinary tract infection in different reports can be probably caused by differences in the geographic areas, the sensitivity of the identification method, the condition of the group (fertile/infertile), other infection accompanied agents, the sample size, and the operator proficiency.

Based on present meta-analysis study the prevalence rates of genital Mycoplasma infection are due to *U. urealyticum* (17.53%), *M. genitalium* (11.33%) and *M. hominis* (9.68%) respectively in Iran which is parallel to Christian Leli (Italy) and Xiaofei (China) results. According to other researcher results, this study shows that the rate of *M. genitalium*, *M. hominis* and *U. urealyticum* infections in women with symptoms of genitourinary tract infection is higher than men with genitourinary tract infection (6.46% Vs 5.4, 7.67% Vs 5.88 and 21.04% Vs 12.13%, respectively). That is in line with kasprzykowska and Mahlangu results. Iranian researches indicated that the prevalence of *M. genitalium*, *U. urealyticum* and *M. hominis* among infertile women (12.73, 19.58 and 10.81%) are higher than fertile women (3%, 10. 85% and 4. 35%), which is similar to Lee (2013) report. However, the prevalence of *M. hominis* and *U. urealyticum* in infertile men (14 and 21.18%) is higher than fertile men (4 and 3%), which is like Lee (2013) result.

According to analysis result, the prevalence of *M. genitalium*, *M. hominis* and *U. urealyticum* in Middle provinces is higher than other provinces in Iran. This may be due to the presence of infertility centers and specialized STD clinics in the capital of the country; Tehran. There are some different diagnostic equipment and facilities to research and attract infertile couples for treatment all over Iran.

## Conclusions

Based on our meta-analysis, the most common Mycoplasma in Iran, in descending order are: *U. urealyticum*, *M. genitalium*, and *M. hominis*. There is statistically significant relationship between couple infertility and *U. urealyticum*, *M. genitalium* and *M. hominis* infections. There is the higher rate of mycoplasmas infection in women than men and their correlated infertilities.

However, obstetricians should consider mycoplasmas infection as a major agent of infertility. Since mycoplasmas are resistant to common antibiotics and the high prevalence of some mycoplasmas like *U. urealyticum*, Iranian physicians should be careful in the treatment of genitourinary tract infections to the possible presence of mycoplasmas agent and the sensitive antibiotics.

Further epidemiological and phylogenetic studies in different provinces will be needed to clarify the exact prevalence and distribution pattern of *U. urealyticum*, *M. genitalium* and *M. hominis* in Iran, and proposed routine screening of the pathogens in patients with infertility.

## Data Availability

Not applicable.

## Abbreviations

M: Mycoplasma
U: Ureaplasma
PCR: polymerase chain reaction
